# The Link Between Serum Copper and Diabetic Kidney Disease: A Plasma Proteomic Perspective

**DOI:** 10.1155/jdr/1950326

**Published:** 2026-03-16

**Authors:** Zhenbang Feng, Jinyu Yu, Junzhuo Wang, Sirui Lu, Weixia Sun, Sensen Su, Wei Liu, Hongzhao Xu

**Affiliations:** ^1^ Center of Oncology, The First Hospital of Jilin University, Changchun, China, jlu.edu.cn; ^2^ Department of Urology, The First Hospital of Jilin University, Changchun, China, jlu.edu.cn; ^3^ Department of Nephrology, The First Hospital of Jilin University, Changchun, China, jlu.edu.cn; ^4^ The First Norman Bethune Clinical College, Jilin University, Changchun, China, jlu.edu.cn

**Keywords:** causal relationship, copper, diabetic kidney disease, mediation analysis, plasma proteomics

## Abstract

**Background:**

Diabetic kidney disease (DKD) is a significant microvascular complication of diabetes, accounting for the majority of cases of end‐stage renal disease. Copper, an essential trace element, has been identified as a factor closely associated with the onset and progression of DKD. However, the precise causal link between serum copper levels and DKD, along with the mechanisms governing their association, remains unclear.

**Methods:**

This study employed Mendelian randomization (MR) analysis based on large‐scale GWAS data to explore the causal relationship between serum copper levels and DKD. Additionally, mediation analysis was conducted using plasma proteomic data, complemented by enrichment analysis to explore the mediating metabolic pathways or signaling pathways through which serum copper may mediate the development of DKD.

**Results:**

MR results demonstrated that elevated serum copper levels significantly increased the risk of DKD (OR = 1.123, *p* < 0.001). Additionally, no evidence of pleiotropy or heterogeneity was detected, which provides further assurance of the reliability of results. Protein mediation analysis identified 10 plasma proteins, including FABP, Netrin‐1, and glutathione S‐transferase A4 as critical mediators in copper‐driven DKD, with mediation effects ranging from 6.42% to 28.02%. Following the identification of these 10 mediator proteins, 11 intermediary pathways that mediated the effect of serum copper levels on the onset of DKD were identified.

**Conclusion:**

This study elucidates the causal effect of copper levels on DKD and reveals the underlying mechanism by which increased serum copper levels lead to the development of DKD, offering novel insights and potential therapeutic targets for DKD prevention and treatment.

## 1. Introduction

Diabetes mellitus (DM) is a chronic metabolic disease marked by hyperglycemia, and it is caused by absolute or relative insufficiency of insulin secretion and impaired utilization [1]. As people’s material living standards improve, the prevalence of DM is increasing year by year [2]. Diabetic kidney disease (DKD) is one of the most prevalent microvascular complications of DM, and epidemiological studies have found that about 40% of DM patients will develop DKD, and DKD is also a significant contributing factor to the development of end‐stage renal disease (ESRD) [3]. ESRD requires renal replacement therapy such as dialysis and renal transplantation, which imposes a heavy economic burden on patients and society. Consequently, DKD has emerged as a global public health concern. At present, there is a dearth of efficacious clinical treatments for DKD. Current approaches are largely symptomatic and supportive, focusing on controlling blood glucose and blood pressure and inhibiting the renin–angiotensin–aldosterone system (RAAS) [4]. It is therefore of great significance to deeply understand the mechanisms underlying the development of DKD and to identify novel intervention targets and treatment modalities.

In recent years, there has been a notable increase in the number of studies examining the significant impact of trace element imbalance on the progression of DKD [5–7]. Among the six essential trace elements, copper plays an indispensable role in maintaining normal physiological functions of the body. It not only participates in the structural composition of many enzymes as a constituent and in the regulation of their activity but also participates in cellular metabolism, energy transduction, oxidative stress, and other important processes [8, 9]. Research indicates that DM affects copper metabolism in the body, and copper can deposit in renal tissues directly, leading to granular degeneration and vacuolar degeneration of the renal tubular epithelium and proximal tubular necrosis [10, 11]. Furthermore, copper has been demonstrated to participate in the pathogenesis of oxidative stress in conjunction with glycosylated proteins [12], which represents a significant contributing factor to the deterioration of renal function. Notably, elevated urinary copper is present in patients with DKD, which may be due to the dissociation of ceruloplasmin–copper complexes during filtration through damaged glomeruli, and the overload of urinary copper allows further progression of DKD [13]. Talaei et al. [14] found a correlation between urinary copper levels and microproteinuria in diabetic patients, revealing that levels of microproteinuria tend to increase in tandem with urinary copper concentrations. Additionally, evidence suggests a relationship between serum zinc levels and copper’s role in the development of DKD, leading to the proposal that the serum Cu/Zn ratio may serve as a marker for assessing DKD progression [15]. However, there is considerable variability in findings across studies [7, 16], with some reporting lower serum copper levels in DKD patients compared to the general population, while others fail to find any significant differences. This adds to the mystery of how copper plays a role in DKD. Such inconsistency further complicates the understanding of the relationship between serum copper and DKD.

Mendelian randomization (MR) is an epidemiological research method that uses genetic variation as an instrumental variable to explore whether exposure factors have a causal effect on health outcomes [17]. Its rising popularity stems from the advantage it offers in avoiding reverse causality and reducing confounding biases that often complicate traditional research methods [18]. A growing number of studies have shown that plasma proteins frequently act as mediators in various diseases [19]. In recent years, as data from genome‐wide association studies (GWAS) of plasma proteomics have been reported, they have opened up the possibility of utilizing MR to delve deeper into the mechanisms behind causality [20, 21]. In this study, based on large‐scale GWAS data, we systematically explored the causal relationship between serum copper levels and DKD using MR methods and further mediated plasma proteomics to delve deeper into the mechanisms by which copper may contribute to the development of DKD. Our findings may facilitate the discovery of novel preventive strategies as well as intervention targets for DKD.

## 2. Methods

### 2.1. Data Sources

Evans et al. [22] published their summary GWAS data on serum copper levels in 2013. In this study, the effects of single nucleotide polymorphisms (SNPs) on blood copper concentrations were analyzed utilizing two adult cohorts from Australia and the United Kingdom, which included a total of 2603 participants. Genotyping was conducted using Illumina chips, and > 2.5 million SNPs were imputed based on HapMap data. The study identified genome‐wide significant associations for serum copper levels, reporting a total of 2,543,646 available SNPs. In another pivotal study, Sun et al. [23] presented summary GWAS data on plasma proteome levels in 2018. This study utilized an expanded version of aptamer‐based multiplex protein assays to construct and query the genetic map of the human plasma proteome. The dataset quantified 3282 plasma proteins in 3301 healthy participants from 25 centers across England as part of the INTERVAL study, a genomic resource encompassing 50,000 blood samples. Genome‐wide testing was performed for 10.6 million imputed autosomal variants. The participants included individuals of European ancestry, both male and female, with SNP counts reaching up to 10,534,735. Additionally, genetic variant data related to health outcomes were sourced from the FinnGen project [24], a large‐scale initiative designed to integrate genetic variation data with extensive health records from over 500,000 Finnish biobank samples. This project is a collaborative effort among Finnish research entities, biobanks, and international industry partners, and all data underwent ethical review. Summary GWAS data for DKD were retrieved from the R9 release of the FinnGen database, published on May 11, 2023. The dataset information is summarized in Table 1, with additional details provided in Table S1. Additionally, we reviewed our analytical process in accordance with the STROBE‐MR checklist to ensure the reliability of the results, with the checklist information available in the Supporting Information section (“STROBE‐MR checklist” file).

**Table 1:** Information on included datasets.

Trait	Case	Year	Author	Gender	Population	NSNP	Sample size
Serum copper level	2603	2013	Evans et al.	Males and females	European	2543646	2603
Serum proteome	3301	2018	Sun et al.	Males and females	European	10534735	3301
Diabetic nephropathy	4111	2023	FinnGen	Males and females	European	20167370	312650

### 2.2. Screening of Instrumental Variables

**Figure 1:**
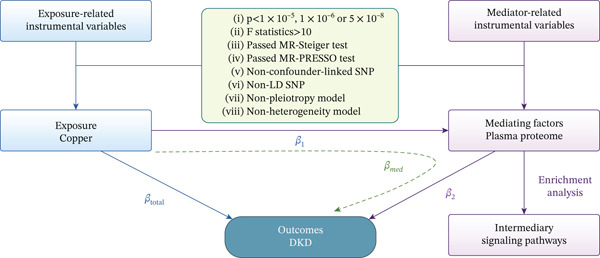
Study design.

The experimental design of this study is illustrated in Figure 1. To conduct MR analysis, certain crucial assumptions regarding instrumental variables must be met. Firstly, the instrumental variable must be significantly associated with the risk factor (relevance assumption). Secondly, the instrumental variable must be independent of any confounding factors (independence assumption). Thirdly, the instrumental variable should influence the outcome only through the risk factor and not via other pathways (exclusion restriction assumption). Based on the selected GWAS summary statistics, we established a series of criteria for screening SNPs. SNPs that were associated with the risk factor at the genome‐wide significance level (*p* value < 5 × 10^−8^) were initially chosen. For a more comprehensive analysis, in cases where no SNPs met this stringent threshold, we applied a more lenient genome‐wide potential significance level (*p* value < 1 × 10^−5^) as the cutoff. SNPs that were in linkage disequilibrium (LD) were removed using a threshold of *r* ^2^ < 0.001 and a window size of > 10,000 kb. Relevant data for SNPs associated with the risk factor were extracted from the summary statistics of the outcome dataset. To ensure the reliability and interpretability of the analysis, ambiguous SNPs and palindromic SNPs were excluded. Palindromic SNPs are defined as those that have identical base sequences on both the forward and reverse strands of DNA. The *F*‐statistic was calculated for each SNP to assess its strength as an instrumental variable; this statistic measures the strength of an SNP in explaining the risk factor. SNPs with an *F* − statistic < 10 were considered weak instruments and were therefore excluded from the analysis [25]. An MR‐PRESSO test was conducted to identify and eliminate SNPs with potential pleiotropy. The MR‐Steiger test was applied to determine the causal direction of each SNP’s effect estimate, and SNPs with incorrect directions were excluded [26]. Finally, PhenoScanner was used to assess the potential associations of each SNP with confounding factors, and SNPs potentially violating the independence assumption were removed. Following these rigorous filtering processes, the remaining SNPs were considered valid instrumental variables.

### 2.3. Mendelian Randomization Analysis

To investigate the causal relationship between exposure and outcomes, we employed a range of robust MR methods grounded in various assumptions. Our analytical toolkit included the inverse variance weighting (IVW), MR‐Egger regression, weighted median (WM), weighted mode, and MR robust adjusted profile score (MR‐RAPS). Among these, IVW stands out as one of the most commonly applied approaches in MR analysis. It combines the ratio estimates of SNPs using a meta‐analytic framework with IVW, thereby yielding an estimate of the influence of risk factors on outcomes [27]. Given its prominence, IVW was the primary method employed in our study. Similar to IVW, MR‐Egger regression [28] incorporates a regression framework but with an intercept term that allows for the evaluation of horizontal pleiotropy. When evidence of pleiotropy is observed, MR‐Egger regression is preferred. Compared to the IVW and MR‐Egger methods, the WM method demonstrates greater robustness [29, 30], making it particularly reliable in scenarios requiring the inclusion of such variants. Furthermore, this study incorporated the newly developed MR‐RAPS method, which directly accounts for the pleiotropic effects of genetic variants using a random‐effects distribution. Compared with traditional MR techniques, this innovative approach offers enhanced robustness. Notably, when the exposure factor comprises only two SNPs as instrumental variables, the IVW and MR‐RAPS methods are employed as the effect estimation methods. These two methods are relatively robust under conditions of finite instrumental variables. IVW provides combined estimates, while MR‐RAPS mitigates potential biases arising from weak instrumental variables to some extent through contour likelihood adjustment. Statistical significance for our findings was established with a threshold of *p* < 0.05. This comprehensive approach, combining several established MR methods, is aimed at supporting more reliable causal inference and reducing potential bias.

### 2.4. Sensitivity Analysis

Pleiotropy, a phenomenon where a single gene influences multiple traits, can be categorized into horizontal and vertical forms. While vertical pleiotropy does not typically affect the validity of study outcomes, horizontal pleiotropy poses a significant threat to the reliability of conclusions and must be addressed. To quantify the extent of horizontal pleiotropy, we employed the MR‐Egger regression method. A *p* value for the MR‐Egger intercept below 0.05 suggests that the instrumental variables are substantially affected by horizontal pleiotropy, casting doubt on the reliability of the findings. In scenarios where pleiotropy is suspected, the MR‐Egger regression serves as the primary analytical approach [30]. In MR analyses, even when all SNPs qualify as valid instrumental variables, heterogeneity among these SNPs may still exist. Such heterogeneity can undermine the robustness of the results, necessitating heterogeneity tests to enhance confidence in the findings. The IVW method was used to calculate heterogeneity among SNPs, with Cochran’s *Q* test applied to assess significance [31]. A *p* value < 0.05 was considered indicative of heterogeneity. According to the MR guidelines, the IVW random‐effects model was employed as the primary analytical approach regardless of whether heterogeneity is detected [30]. Additionally, sensitivity analyses included leave‐one‐out analyses and the construction of funnel plots. A global MR‐Steiger test was conducted to validate the overall direction of causality. Lastly, statistical power calculations were performed to ascertain the reliability of negative findings.

### 2.5. Proteomic Mediation Analysis of Metabolic and Signaling Pathways

The mediation effects in this study were quantified within a two‐step MR framework on a unified scale. Specifically, we first estimated the total effect of serum copper on DKD, expressed on the log(OR) scale, and separately obtained the effect of serum copper on each candidate protein (*β*1) and the effect of each protein on DKD (*β*2). The indirect effect (average causal mediation effect [ACME]) was defined as *β*_indirect = *β*1×*β*2, while the direct effect (average direct effect [ADE]) was defined as *β*_direct = *β*_total − *β*_indirect. The mediation proportion was calculated as *β*_indirect/*β*_total. To quantify uncertainty in the indirect effect and mediation proportion, a parametric bootstrap approach was applied. We assumed that the estimates of *β*1 and *β*2 approximately followed normal distributions with means equal to their point estimates and variances equal to their squared standard errors. We then generated 1000 random draws for *β*1 and *β*2 and, in each iteration, calculated *β*1×*β*2 and the corresponding mediation proportion, thereby constructing empirical distributions. Based on these empirical distributions, we reported point estimates and 95% confidence intervals (CIs) (derived using the percentile method) for both the indirect effect and the mediation proportion and interpreted the statistical significance of the indirect effect on the log(OR) scale. To avoid bias arising from scale inconsistency, all mediation‐related quantities were computed on the log(OR) scale and converted to the OR scale only when presentation was required. In addition, proteins with implausible effect directions, where the indirect effect was inconsistent with the total effect, were also excluded. Pathway enrichment analysis was conducted on the identified mediating proteins utilizing the Reactome database [32], a peer‐reviewed pathway repository that offers intuitive bioinformatics tools for the visualization, interpretation, and analysis of biological pathways, thereby supporting fundamental research and genomic analysis. Protein names were input into the Analysis Tools section of Reactome, using the human database (*Homo sapiens*, Code 9606) for enrichment analysis. Additionally, the STRING database (http://string-db.org) was employed to investigate the protein–protein interaction network, with a high confidence threshold (0.7) established to retain only the most reliable protein interactions.

### 2.6. Statistical Software and Visualization

To facilitate visualization of the results, scatter plots were generated for each SNP, illustrating the relationship between the exposure effects and the outcome effects, accompanied by regression curves to demonstrate causal estimates. A significance heatmap for MR analysis was meticulously crafted to present the results. Funnel plots were employed to evaluate potential directional pleiotropy and assess the distribution of the data. The final causal estimates were utilized to generate forest plots, which presented the results for each SNP and the overall MR analysis. All statistical analyses in this study were conducted using R software (Version 4.2.3) and the R packages TwoSampleMR, MR‐PRESSO, and mr.raps.

## 3. Results

### 3.1. Characteristics of the Dataset

Based on the information from the dataset sources, there is no sample overlap in the MR analysis of this study. The SNPs that exhibited a strong correlation with serum copper levels were predominantly mapped to Chromosome 1 (Figure 2a). Among these, SNPs rs2769264 and rs1175550 reached genome‐wide significance, with mutations in these SNPs consistently promoting elevated serum copper levels (Figure 2b). In contrast, the SNPs strongly associated with DKD were mainly located on Chromosome 6 (Figure 3a). Among the top five genome‐wide significant SNPs, rs9273363, rs9270891, rs59377618, rs564782870, and rs11967629 were identified. Mutations in these SNPs were shown to significantly contribute to the development of DKD (Figure 3b). Details regarding the SNPs included in the MR study are presented in Table S2.

Figure 2Characteristics and association results of the serum copper level dataset. (a) Manhattan plot of GWAS data for serum copper levels. (b) Two SNPs associated with serum copper levels at genome‐wide significance (after LD pruning). (c) Estimation of the causal relationship between serum copper levels and diabetic nephropathy. (d) Causal relationship results between serum copper levels and the five most significant serum proteins. (e) Effect–significance scatter plot of serum copper levels on the serum proteome, with significantly mediating proteins labeled.

**Figure:**
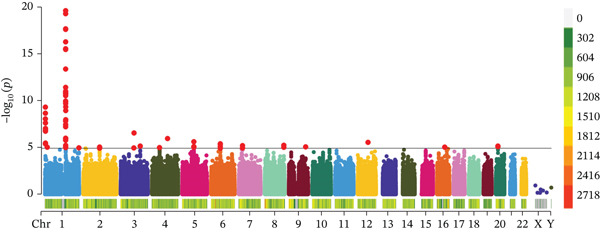
(a)

**Figure:**
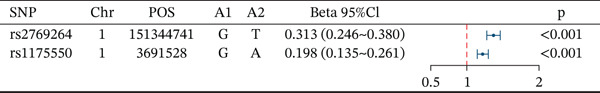
(b)

**Figure:**
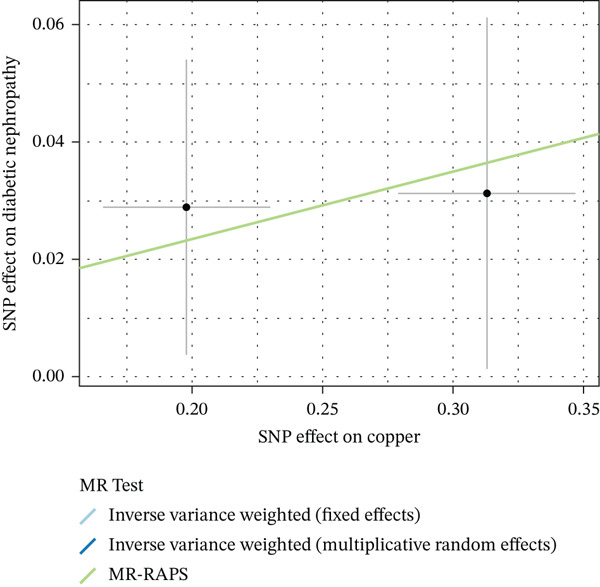
(c)

**Figure:**
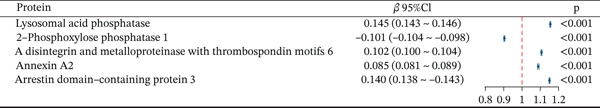
(d)

**Figure:**
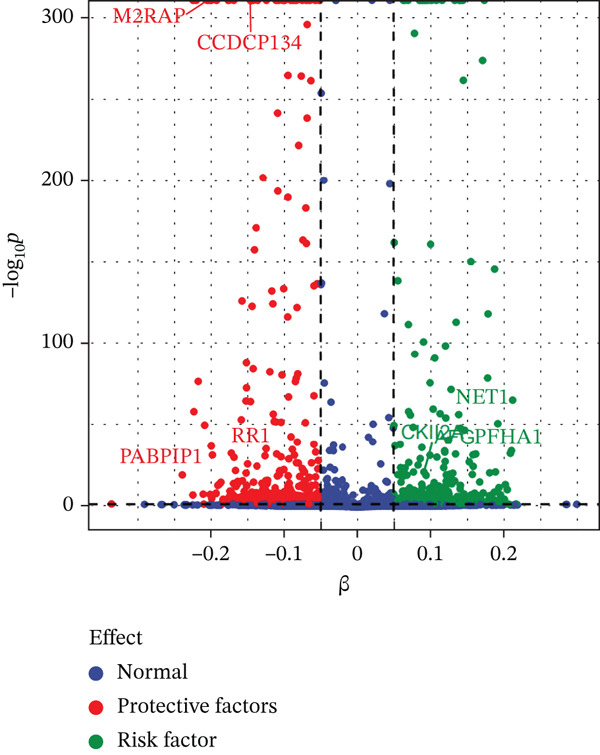
(e)

Figure 3Characteristics and association results of the diabetic nephropathy dataset. (a) Manhattan plot of GWAS data for the diabetic nephropathy dataset. (b) The top five SNPs significantly associated with diabetic nephropathy at genome‐wide significance (after LD pruning). (c) Protein–protein interaction results of significant serum proteins contributing to diabetic nephropathy, clustered by *k*‐means and displaying only the group with the largest number of proteins. (d) The five most significant serum proteins contributing to diabetic nephropathy development. (e) Effect–significance scatter plot of the serum proteome on diabetic nephropathy.

**Figure:**
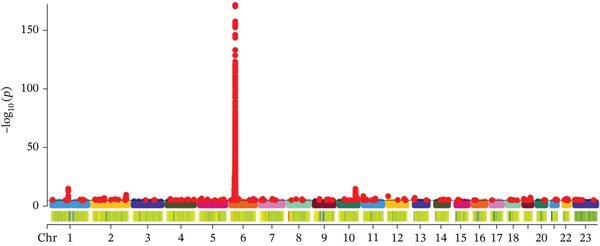
(a)

**Figure:**
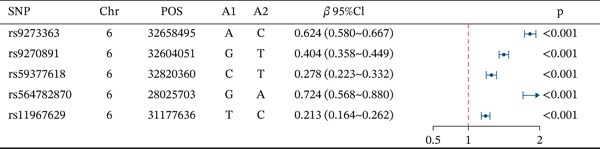
(b)

**Figure:**
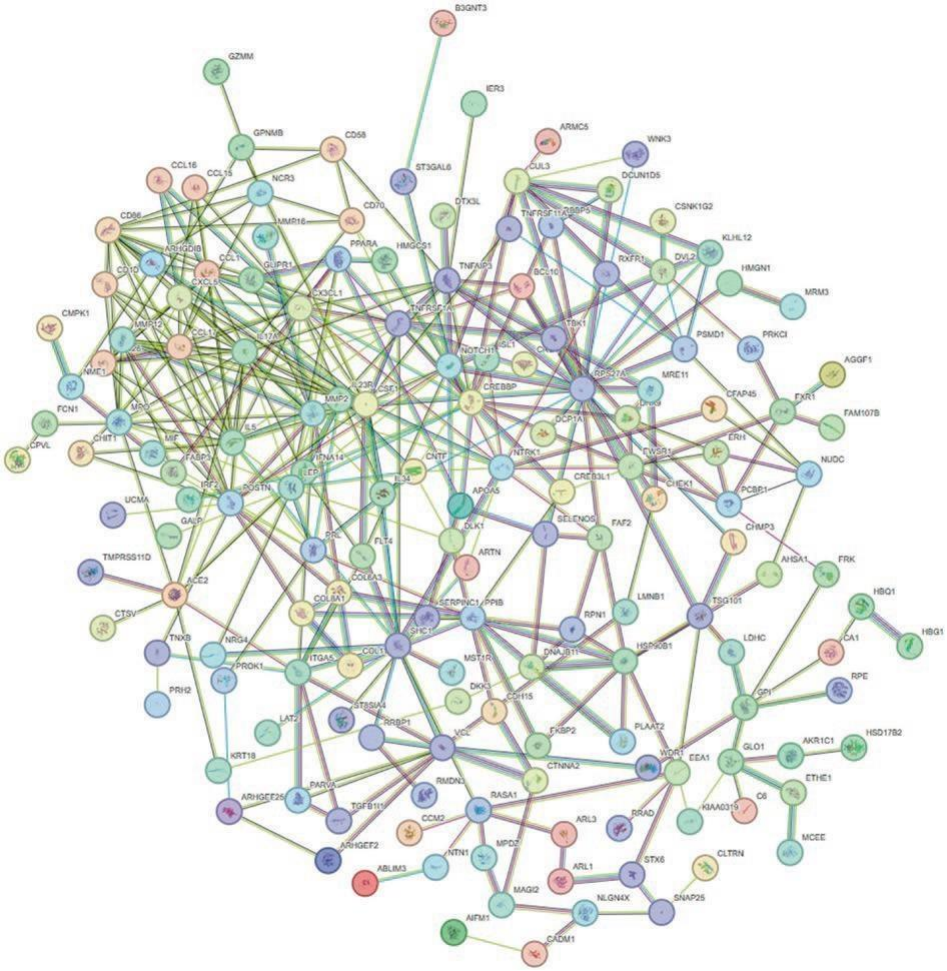
(c)

**Figure:**
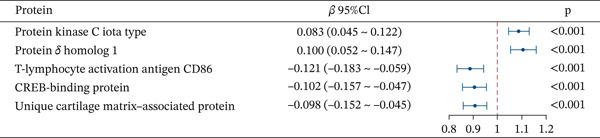
(d)

**Figure:**
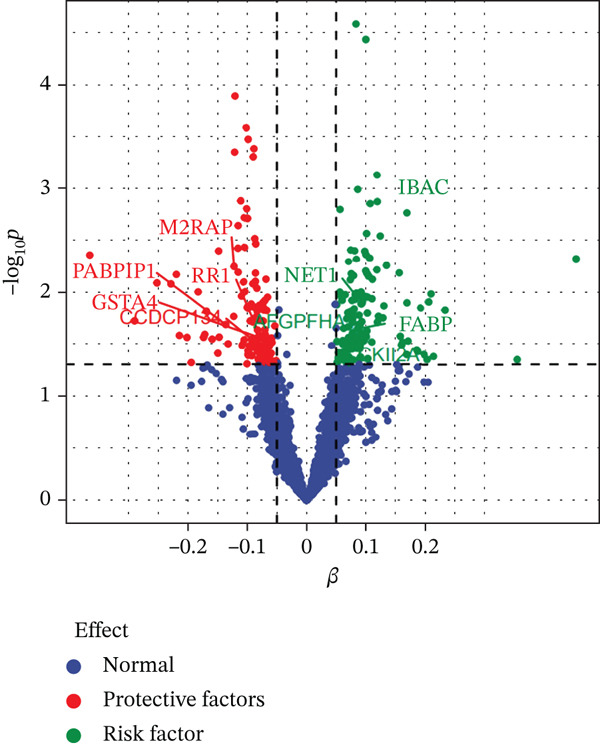
(e)

### 3.2. Selection of Instrumental Variables

In the MR analysis, we initially screened two SNPs associated with serum copper levels. No evidence of weak instrumental variables was detected. The *F*‐statistics for all instrumental variables exceeded the conventional threshold of 10 (minimum = 19.4, median = 29.2), indicating sufficient instrument strength. Detailed statistics for each SNP, including individual *F*‐statistics and association *p* values, are provided in Table S2. The MR‐PRESSO test did not reveal evidence of horizontal pleiotropy among these SNPs, and the MR‐Steiger test provided no evidence of violations in the assumed causal direction. Additionally, we did not identify any SNPs associated with potential confounding factors. Consequently, these two eligible SNPs were included in our analysis. For the mediation MR analysis, we initially screened a total of 111,652 SNPs associated with the exposure. No weak instrumental variables were identified. A total of 12,138 SNPs were excluded due to missing data in the outcome database, and 13,769 SNPs were removed as ambiguous SNPs or palindromic SNPs during dataset harmonization. The MR‐PRESSO test did not reveal any SNPs with horizontal pleiotropy, and the MR‐Steiger test also did not identify any SNPs with incorrect causal directions. Following the Bonferroni correction, 675 SNPs directly associated with the outcome were excluded. Ultimately, we conducted 6565 batches of MR analyses, incorporating a total of 85,070 eligible SNPs. Across each individual MR analysis, the median number of included SNPs was 10, with an interquartile range (IQR) of 22.

### 3.3. Proteomic‐Based Mediation Analysis

In the MR analysis, a per 1 SD increase in serum copper levels was associated with a 12.3% higher risk of DKD (OR [95% CI]: 1.123 [1.076–1.173], *p* < 0.001), with no evidence of heterogeneity or pleiotropy detected (Table 2, Figure 2c). In the mediation MR analysis, a threshold of *p* < 0.05 was applied. We identified 937 plasma proteins influenced by serum copper levels (Figure 2d,e) and 253 proteins showing causal associations with the development and progression of DKD (Figure 3c;e). By intersecting the results of these two mediation steps and ensuring directionality (i.e., the direction in which elevated serum copper levels cause changes in protein levels and the direction in which altered protein levels lead to changes in DKD risk), 10 plasma proteins were ultimately identified as mediators, with mediation proportions ranging from 6.42% to 28.02% (Table 2 reports the mediation effects, mediation proportions, and their corresponding CIs). As shown in the forest plots and scatter plots in Figure 4a,b, poly(A)‐binding protein–interacting protein 1 (PABP1) exhibited the highest mediating effect (mediating OR [95% CI]: 1.033 [1.013–1.054], *p* = 0.001), suggesting it may serve as a potential key intermediary in the pathway linking copper levels to the pathogenesis of DKD. Detailed information is presented in Table 2. Table S3 presents the MR results across all cohorts, with Pre‐batch 2 corresponding to the serum copper‐to‐proteome analyses and Pre‐batch 3 to the proteome‐to‐DKD analyses. Tables S4–S6 provide detailed results of the sensitivity analyses, and an overview of the effect estimates for the mediator proteins is summarized in Table S7.

**Table 2:** Plasma proteins with mediating effects.

Mediator	Shortage	Total effect		Step 1 mediating effect		Step 2 mediating effect		Mediating effect		Direction	Mediating rate	
		OR (95% CI)	*p*	OR (95% CI)	*p*	OR (95% CI)	*p*	OR (95% CI)	*p*		Est.	95% CI
Angiogenic factor with G patch and FHA domains 1	AFGPFHA1	1.123 (1.076–1.173)	< 0.001	1.176 (1.145–1.208)	< 0.001	1.082 (1.014–1.154)	0.017	1.013 (1.006–1.020)	< 0.001	True	10.97%	5.2%–17.1%
Coiled‐coil domain‐containing protein 134	CCDCP134	1.123 (1.076–1.173)	< 0.001	0.864 (0.861–0.867)	< 0.001	0.925 (0.861–0.994)	0.033	1.011 (1.006–1.017)	< 0.001	True	9.81%	5.2%–14.5%
Casein kinase II 2‐alpha ^′^:2‐beta heterotetramer	CKII2A	1.123 (1.076–1.173)	< 0.001	1.096 (1.075–1.116)	< 0.001	1.094 (1.006–1.190)	0.036	1.008 (1.000–1.016)	0.037	True	7.05%	0.9%–13.7%
Fatty acid–binding protein, heart	FABP	1.123 (1.076–1.173)	< 0.001	1.181 (1.104–1.262)	< 0.001	1.090 (1.012–1.173)	0.023	1.014 (1.001–1.027)	0.029	True	12.26%	0.9%–23.0%
Glutathione S‐transferase A4	GSTA4	1.123 (1.076–1.173)	< 0.001	0.911 (0.890–0.933)	< 0.001	0.923 (0.859–0.991)	0.027	1.008 (1.001–1.014)	0.017	True	6.42%	0.9%–12.0%
Inhibin beta A chain:inhibin beta B chain heterodimer	IBAC	1.123 (1.076–1.173)	< 0.001	1.198 (1.099–1.305)	< 0.001	1.127 (1.047–1.212)	0.001	1.022 (1.004–1.040)	0.018	True	18.47%	3.4%–33.8%
Melanocortin‐2 receptor accessory protein	M2RAP	1.123 (1.076–1.173)	< 0.001	0.815 (0.810–0.820)	< 0.001	0.885 (0.811–0.965)	0.006	1.025 (1.014–1.036)	< 0.001	True	21.56%	12.0%–30.5%
Netrin‐1	NET1	1.123 (1.076–1.173)	< 0.001	1.149 (1.130–1.169)	< 0.001	1.086 (1.019–1.156)	0.011	1.011 (1.006–1.017)	< 0.001	True	9.82%	5.2%–14.5%
Polyadenylate‐binding protein–interacting protein 1	PABPIP1	1.123 (1.076–1.173)	< 0.001	0.787 (0.748–0.829)	< 0.001	0.872 (0.777–0.979)	0.021	1.033 (1.013–1.054)	0.001	True	28.02%	11.1%–45.3%
Relaxin receptor 1	RR1	1.123 (1.076–1.173)	< 0.001	0.896 (0.880–0.912)	< 0.001	0.921 (0.857–0.989)	0.023	1.009 (1.003–1.015)	0.004	True	7.81%	2.6%–12.8%

*Note:* The mediation effect was derived by combining the first‐step and second‐step mediation effects. The indirect effect can be obtained by subtracting the direct effect from the total effect.

Figure 4Schematic representation of mediating serum proteins and activated pathways. (a) Schematic representation of direct effects, indirect effects, and mediating effects of significant mediating proteins. (b) Scatter plot of mediation effects for the first and second steps. (c) Schematic diagram of pathway activation.

**Figure:**
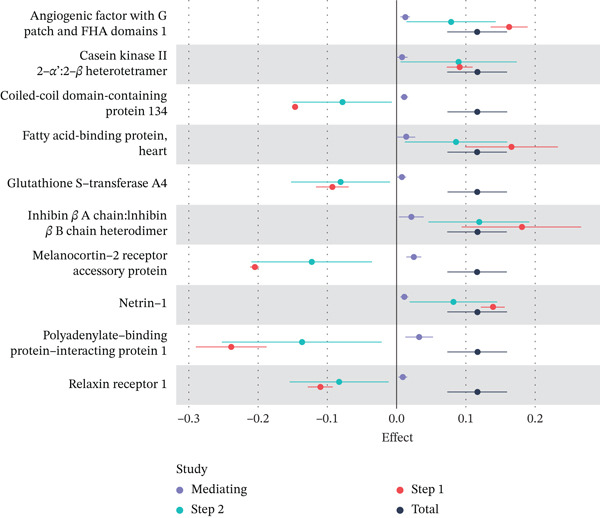
(a)

**Figure:**
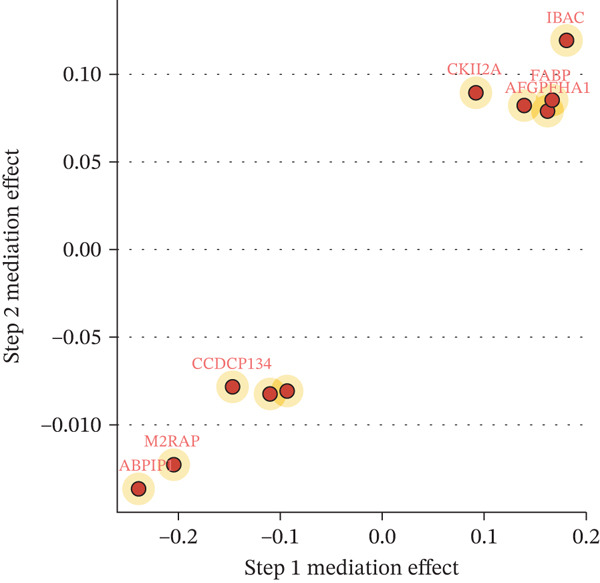
(b)

**Figure:**
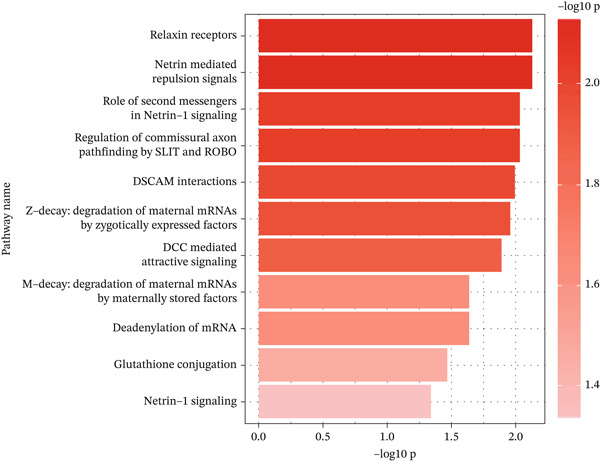
(c)

### 3.4. Activated Metabolic and Signaling Pathways

As illustrated in Table 3 and Figure 4c, our enrichment analysis revealed 11 intermediary pathways through which serum copper is implicated in the progression of diabetes. The five most significant pathways identified are as follows: Netrin‐mediated repulsion signals (reactions found: 4/4, reactions ratio: 2.79*E* − 04, *p*: 0.007), Relaxin receptors (reactions found: 1/4, reactions ratio: 2.79*E* − 04, *p*: 0.007), Role of second messengers in Netrin‐1 signaling (reactions found: 2/4, reactions ratio: 2.79*E* − 04, *p*: 0.009), Regulation of commissural axon pathfinding by SLIT and ROBO (reactions found: 2/5, reactions ratio: 3.48*E* − 04, *p*: 0.009), and DSCAM interactions (reactions found: 1/6, reactions ratio: 4.18*E* − 04, *p*: 0.010).

**Table 3:** Activated metabolic or signal transduction pathways.

Pathway name	Reactions found	Reactions ratio	Entities found	Entities ratio	*p*value	Mapped entities
Netrin‐mediated repulsion signals	4/4	2.79*E* − 04	1/8	6.82*E* − 04	0.007	Netrin‐1
Relaxin receptors	1/4	2.79*E* − 04	1/8	6.82*E* − 04	0.007	Relaxin receptor 1
Role of second messengers in Netrin‐1 signaling	2/4	2.79*E* − 04	1/10	8.53*E* − 04	0.009	Netrin‐1
Regulation of commissural axon pathfinding by SLIT and ROBO	2/5	3.48*E* − 04	1/10	8.53*E* − 04	0.009	Netrin‐1
DSCAM interactions	1/6	4.18*E* − 04	1/11	9.38*E* − 04	0.010	Netrin‐1
Z‐decay: degradation of maternal mRNAs by zygotically expressed factors	4/4	2.79*E* − 04	1/12	1.02*E* − 03	0.011	Polyadenylate‐binding protein–interacting protein 1
DCC‐mediated attractive signaling	12/12	8.36*E* − 04	1/14	1.19*E* − 03	0.013	Netrin‐1
Deadenylation of mRNA	4/4	2.79*E* − 04	1/25	2.13*E* − 03	0.023	Polyadenylate‐binding protein–interacting protein 1
M‐decay: degradation of maternal mRNAs by maternally stored factors	2/7	4.88*E* − 04	1/25	2.13*E* − 03	0.023	Polyadenylate‐binding protein–interacting protein 1
Glutathione conjugation	1/12	8.36*E* − 04	1/37	3.16*E* − 03	0.034	Glutathione S‐transferase A4
Netrin‐1 signaling	22/37	2.58*E* − 03	1/50	4.26*E* − 03	0.046	Netrin‐1

## 4. Discussion

Copper is an essential trace element in organisms, serving as a key component in enzyme synthesis and participating in cellular metabolism and oxidative stress responses [8, 9]. It is notably concentrated in the kidneys, which are pivotal in copper homeostasis [33], but elevated systemic copper levels can induce nephrotoxicity, including vacuolar degeneration of renal tubular cells [10–13]. In diabetic patients, copper metabolism is often disrupted [11], and this dysregulation is increasingly recognized as a significant contributor to the progression of ESRD, with approximately 40% of diabetic patients developing DKD [3]. Current therapeutic options are limited to renal replacement therapy and symptomatic supportive care [4]. Therefore, exploring the potential role of copper in the development of DKD is important for future clinical interventions in DKD. In this study, based on large‐scale GWAS data, we systematically explored the causal relationship between serum copper levels and DKD using MR methods and found that an increase in serum copper levels would lead to an increased risk of DKD. Notably, our study analyzed total serum copper levels, whereas the majority of serum copper exists in a bound form with ceruloplasmin. As an acute‐phase reactant protein, ceruloplasmin levels may be influenced by various factors, including systemic inflammation and metabolic status [34]. This suggests that alterations in total serum copper levels may partially reflect inflammatory states rather than solely changes in copper homeostasis. However, as ceruloplasmin‐bound copper constitutes the primary copper reservoir in the circulatory system, its levels directly influence copper’s bioavailability and subsequent tissue distribution and biological effects. Therefore, the findings of this study hold significant clinical implications, suggesting that targeted interventions, such as rational copper supplementation and dietary adjustments, could be employed to mitigate the risk of DKD in high‐risk populations.

Hyperglycemia is known to be the primary factor in DKD development [3]. Clinical studies have noted that renal damage could occur even in DM patients with good glycemic control [4], suggesting that numerous complex mechanisms are involved in the development of DKD, including not only an increase in blood glucose levels but also excessive activation of RAAS, abnormalities in glucose internalization and metabolism, and so on. Previous MR studies on copper and DM found that serum copper levels did not correlate with DM [35–37]. But interestingly, our MR study found that increased serum copper levels led to an increased risk of DKD, suggesting that serum copper may contribute to DKD progression through renal damage pathways independent of blood glucose levels. Given the pivotal role of plasma proteins in disease pathogenesis as mediators, we conducted a plasma proteome mediation analysis to elucidate the mechanisms underlying the heightened risk of DKD associated with increased serum copper levels. Our analysis identified 10 plasma proteins that may play important roles in the underlying mechanisms, including PABP1, fatty acid–binding protein (FABP), Netrin‐1, and glutathione S‐transferase A4 (GSTA4). Of these, PABP1 exhibited the highest mediation proportion. PABP1 is a translational regulator involved in mRNA stability and protein synthesis initiation [38]. Although its direct role in the pathogenesis of DKD remains uncharacterized, its central function in translational control provides a plausible link to the disease mechanism. In the diabetic environment, cellular stress induced by hyperglycemia and metabolic dysregulation, such as oxidative stress and endoplasmic reticulum stress, can adversely affect mRNA translation [39]. Consequently, PABP1 may contribute to the progression of DKD by regulating the translation of proteins associated with renal inflammation, fibrosis, or podocyte injury.

In the present study, we have identified a significant relationship between elevated serum copper levels and increased levels of FABP. Notably, heightened FABP levels are associated with the role of copper in promoting lipolysis [40]. FABP may be instrumental in facilitating the cellular uptake, incorporation, and translocation of fatty acids [41]. Its levels rise concomitantly with enhanced lipolysis, and increased FABP1 has been correlated with insulin resistance and the onset of DM [42]. This elevation in FABP levels may consequently raise the risk of developing diabetes and associated complications, including DKD. In addition to this, FABP3 has been implicated in the induction of mitochondrial autophagy, a process associated with apoptosis [43]. Elevated concentrations of FABP3 have been observed in the urine of patients with DKD [44], suggesting that increased FABP levels contribute to the progression of this condition. In addition to FABP, we observed that serum levels of Netrin‐1 also rise in response to increased copper concentrations in the body. This elevated Netrin‐1 could be linked to direct renal damage caused by high copper levels [10, 11], as it can be induced by the damaged renal tubular epithelium [45]. Meanwhile, Ay et al. [46] found that significant increases in serum Netrin‐1 levels have also been documented in DKD patients with microproteinuria, demonstrating a correlation between Netrin‐1, albuminuria, and estimated eGFR. These findings further implicate elevated Netrin‐1 in the progression of DKD. Moreover, elevated serum copper levels have been associated with decreased concentrations of certain plasma proteins, notably GSTA4. GST is an antioxidant consisting of a family of multigene proteins involved in the metabolism of many pathogenic proelectrophilic substrates and protects cells from oxidative stress [47], of which GSTA4 is one isoform. When the copper level is too high, it can increase the level of oxidative stress in cells, and the generated ROS may drive the development of renal fibrosis by triggering lipid peroxidation (LPO) [48, 49]. One prominent byproduct of the LPO process is 4‐hydroxynonenal (4‐NHE), which has a tendency to accumulate within cells and is closely linked to the induction of endoplasmic reticulum stress in renal tubular cells [50, 51]. GSTA4 exhibits significant catalytic activity toward 4‐NHE [52]. Research indicates that GSTA4 may mitigate renal fibrosis by lowering the accumulation of 4‐NHE [53]. However, increased levels of ROS are known to impair GST activity [54], potentially leading to a depletion of GSTA4, which serves as an antioxidant. Consequently, the reduction in GSTA4 levels in response to elevated copper concentrations might contribute to an enhanced risk of renal fibrosis progression. Notably, fatty acid–binding protein 5 (FABP5) has been implicated in mediating the production of 4‐NHE via LPO [55], suggesting a potential interplay between FABP5 and GSTA4 in the pathogenesis of DKD. Notably, these identified mediator proteins are all dynamic biomarkers, potentially exhibiting heterogeneity in DKD. Our MR framework estimates the genetic effects of these traits, providing evidence for causality and mitigating reverse causality bias. However, this approach does not capture how these causal roles may evolve or vary in intensity throughout different disease stages. Future longitudinal studies with serial measurements are needed to delineate their stage‐specific contributions to DKD pathogenesis.

To further investigate the mechanism of elevated copper levels in the development of DKD, we identified 11 pathways involved in the enrichment analysis, including Netrin‐1 signaling, glutathione conjugation, and so on. As mentioned above, the levels of Netrin‐1 increase concomitantly with elevated copper levels, and numerous studies have proposed Netrin‐1 as a potential biomarker for kidney injury [46]. In‐depth investigations into Netrin‐1 have revealed its association with the MAPK pathway [56], wherein elevated levels of Netrin‐1 can attenuate the production of inflammatory prostaglandin E2 (PGE2) by inhibiting the nuclear factor kappa B (NF‐*κ*B)–mediated expression of cyclooxygenase‐2 (COX‐2) in renal tubular epithelial cells. Furthermore, Netrin‐1 has been reported to enhance albumin uptake through phosphoinositide 3‐kinase (PI3K) and extracellular signal–regulated kinase (ERK) signaling pathways, which could contribute to the mitigation of chronic kidney injury in diabetic patients [57]. In addition, research by Tak et al. [58] suggested the absence of protective effects associated with Netrin‐1‐mediated inflammatory responses in Adora2b knockout mice, thereby implying a potential link between Netrin‐1 signaling and Adora2b. Additionally, the mechanism of glutathione conjugation has been implicated in the development of DKD induced by copper exposure. Glutathione conjugation has been recognized as a detoxification reaction, where glutathione can be associated with a nucleophilic attack on an electrophilic substrate, thus providing protection to the cell [59]. Copper, identified as an electrophilic agent, interacts with glutathione, which is instrumental in regulating both the influx and efflux of copper ions within cells. Notably, glutathione serves as the primary receptor that binds to copper immediately upon its cellular entry [60, 61]. Through this interaction, glutathione could help mitigate copper‐induced oxidative stress, potentially playing a protective role in tissues. Among them, GST is involved in the process of catalytic coupling [62, 63]. Specifically, GSTA4 has been suggested to contribute to the attenuation of renal fibrosis by degrading 4‐NHE [53]. However, it is noteworthy that GSTA4 levels diminish in response to elevated copper concentrations. Therefore, further exploration of the relationship between high copper levels and the coupling efficiency of glutathione is warranted to better understand the implications for cellular protection and disease pathogenesis.

This study acknowledges several limitations that warrant consideration. Firstly, the GWAS data concerning serum copper levels were obtained from two cohorts of adults located in Australia and the United Kingdom. Previous research has indicated variability in serum copper levels across different populations [7, 16], which may be attributed to factors such as ethnicity, diet, and local environmental conditions. Consequently, the generalizability of our findings might be restricted. Secondly, our main MR analysis and the first‐step mediation MR analysis were limited by the availability of only two SNPs strongly associated with serum copper levels. This restricted the application of several analytical methods (such as MR‐Egger, WM, and weighted mode) and the ability to comprehensively assess potential pleiotropy or instrument validity. Although robust methods suitable for a few instruments were also employed, this constraint may affect the validation capacity of our findings. Thirdly, as an exploratory study, multiple testing correction was not applied to avoid overlooking potentially biologically significant associations with smaller effect sizes. Consequently, findings at the protein level may carry a risk of false positives. Thus, these discoveries should be interpreted with caution and require validation in independent cohorts or further experimental studies. Fourthly, the GWAS data on plasma proteome levels were derived from a population of European ancestry, further limiting the applicability of our results to the broader population. Additionally, our analysis relied on publicly available summary statistics, and raw clinical outcome data for individual participants were not accessible. It is also unable to distinguish between the effects of ceruloplasmin‐bound copper and free copper at the genetic level. These constraints hindered our ability to conduct further population stratification analyses by disease stage or clinical subgroup and also constrained further exploration of the underlying mechanisms. Lastly, although we conducted a comprehensive examination of various plasma proteins and pathways involved in DKD, we did not experimentally validate this, and the specific causal effects of this need to be explored in further studies.

## 5. Conclusions

In this study, we utilized a two‐step proteomic‐mediated MR approach to investigate the causal role of serum copper in DKD and its potential mechanisms. Our analysis revealed a positive association between genetically predicted elevated serum copper levels and an increased risk of DKD. Furthermore, we identified 10 plasma proteins, including PABP1 and Netrin‐1, as potential mediators of this relationship, with enrichment analysis highlighting related biological pathways such as Netrin‐mediated repulsion signals. These findings suggest plausible molecular links between copper homeostasis and DKD pathogenesis. However, given the limitations of the analytical methods employed in this study, including the restricted number of instrumental variables for serum copper and the exploratory nature of the proteomic screening, future research is required in independent cohorts and experimental models to validate these associations and elucidate their precise biological mechanisms.

**Nomenclature**
COX‐2: cyclooxygenase‐2
DKD: diabetic kidney disease
DM: diabetes mellitus
ERK: extracellular signal–regulated kinase
ESRD: end‐stage renal disease
FABP: fatty acid–binding protein
FABP5: fatty acid–binding protein 5
GSTA4: glutathione S‐transferase A4
GWAS: genome‐wide association study
IVW: inverse variance weighting
LPO: lipid peroxidation
MR: Mendelian randomization
MR‐RAPS: MR robust adjusted profile score
NF‐*κ*B: nuclear factor kappa B
PGE2: prostaglandin E2
PI3K: phosphoinositide 3‐kinase
RAAS: renin–angiotensin–aldosterone system
SNPs: single nucleotide polymorphisms
WM: weighted median

## Author Contributions

Z.F. conceived and designed the research and conducted the analysis. J.Y. and J.W. wrote the original draft of the manuscript. S.L. and W.S. curated and analyzed the data. S.S. and W.L. contributed to manuscript editing. H.X. supervised the study and reviewed the manuscript. Z.F. and J.Y. contributed equally to this work.

## Funding

This study was supported by the Natural Science Foundation of Jilin Province—Talent Specialization‐Youth Growth Science and Technology Project (No. 20240602091RC).

## Disclosure

All authors agree to publish. All authors made substantial contributions to the study and approved the final version.

## Conflicts of Interest

The authors declare no conflicts of interest.

## Supporting Information

Additional supporting information can be found online in the Supporting Information section.

## Supporting information


**Supporting Information 1** STROBE‐MR checklist.

**Supporting Information 2** Table S1: Dataset information included in MR studies. Table S2: SNP information included in MR studies. Table S3: MR results for all batches. Table S4: MR‐Steiger test results for SNPs. Table S5: Cochran’s *Q* test results for all batches. Table S6: Results of pleiotropic tests for all batches. Table S7: Results of two‐step mediation analysis.

## Data Availability

The data that support the findings of this study are available in the Supporting Information section of this article.

## References

[1] Petersmann A. , Müller-Wieland D. , Müller U. A. , Landgraf R. , Nauck M. , Freckmann G. , Heinemann L. , and Schleicher E. , Definition Classification and Diagnosis of Diabetes Mellitus, Experimental and Clinical Endocrinology & Diabetes. (2019) 127, no. S 01, S1–S7, 10.1055/a-1018-9078. 31860923

[2] Iminger-Finger I. , Kargul J. , and Laurent G. J. , Diabetes: Present and Future, International Journal of Biochemistry & Cell Biology. (2017) 88, 10.1016/j.biocel.2017.06.003, 2-s2.0-85020790481. 28600145

[3] Alicic R. Z. , Rooney M. T. , and Tuttle K. R. , Diabetic kidney disease: challenges, progress, and possibilities, Clinical Journal of the American Society of Nephrology. (2017) 12, no. 12, 2032–2045, 10.2215/CJN.11491116, 2-s2.0-85020046943. 28522654 PMC5718284

[4] Thomas M. C. , Brownlee M. , Susztak K. , Sharma K. , Jandeleit-Dahm K. A. M. , Zoungas S. , Rossing P. , Groop P.-H. , and Cooper M. E. , Diabetic Kidney Disease, Nature Reviews Disease Primers. (2015) 1, no. 1, 10.1038/nrdp.2015.18, 2-s2.0-85017272654. PMC7724636 27188921

[5] Bahrampour N. , Mirzababaei A. , Abaj F. , Hosseininasab D. , Clark C. C. T. , and Mirzaei K. , The Association Between Dietary Micronutrient Patterns and Odds of Diabetic Nephropathy: A Case–Control Study, Food Science & Nutrition. (2023) 11, no. 6, 3255–3265, 10.1002/fsn3.3306. 37324888 PMC10261793

[6] Ming J. , Sana S. R. G. L. , and Deng X. , Identification of Copper-Related Biomarkers and Potential Molecule Mechanism in Diabetic Nephropathy, Frontiers in Endocrinology. (2022) 13, 978601, 10.3389/fendo.2022.978601. 36329882 PMC9623046

[7] Prabodh S. , Prakash D. S. R. S. , Sudhakar G. , Chowdary N. V. S. , Desai V. , and Shekhar R. , Status of Copper and Magnesium Levels in Diabetic Nephropathy Cases: A Case-Control Study From South India, Biological Trace Element Research. (2011) 142, no. 1, 29–35, 10.1007/s12011-010-8750-x, 2-s2.0-79959553077. 20552294

[8] Brewer G. , Copper in Medicine, Current Opinion in Chemical Biology. (2003) 7, no. 2, 207–212, 10.1016/S1367-5931(03)00018-8, 2-s2.0-0037399007. 12714053

[9] Uriu-Adams J. Y. and Keen C. L. , Copper, Oxidative Stress, and Human Health, Molecular Aspects of Medicine. (2005) 26, no. 4-5, 268–298, 10.1016/j.mam.2005.07.015, 2-s2.0-24044481966. 16112185

[10] Alak G. , Yeltekin A. Ç. , Uçar A. , Parlak V. , Türkez H. , and Atamanalp M. , Borax Alleviates Copper-Induced Renal Injury via Inhibiting the DNA Damage and Apoptosis in Rainbow Trout, Biological Trace Element Research. (2019) 191, no. 2, 495–501, 10.1007/s12011-018-1622-5, 2-s2.0-85059588145. 30612301

[11] Gembillo G. , Labbozzetta V. , Giuffrida A. E. , Peritore L. , Calabrese V. , Spinella C. , Stancanelli M. R. , Spallino E. , Visconti L. , and Santoro D. , Potential Role of Copper in Diabetes and Diabetic Kidney Disease, Metabolites. (2022) 13, no. 1, 10.3390/metabo13010017. PMC9866181 36676942

[12] Eaton J. W. and Qian M. W. , Interactions of Copper With Glycated Proteins: Possible Involvement in the Etiology of Diabetic Neuropathy, Molecular and Cellular Biochemistry. (2002) 234, no. 1, 135–142, 10.1023/A:1015988817587, 2-s2.0-4243704850. 12162426

[13] Ito S. , Fujita H. , Narita T. , Yaginuma T. , Kawarada Y. , Kawagoe M. , and Sugiyama T. , Urinary Copper Excretion in Type 2 Diabetic Patients With Nephropathy, Nephron. (2001) 88, no. 4, 307–312, 10.1159/000046013, 2-s2.0-0034939396. 11474224

[14] Talaei A. , Jabari S. , Bigdeli M. , Farahani H. , and Siavash M. , Correlation Between Microalbuminuria and Urinary Copper in Type Two Diabetic Patients. *Indian Journal of* , Indian journal of endocrinology and metabolism. (2011) 15, no. 4, 10.4103/2230-8210.85586. PMC3193781 22029003

[15] Takao T. , Yanagisawa H. , Suka M. , Yoshida Y. , Onishi Y. , Tahara T. , Kikuchi T. , Kushiyama A. , Anai M. , Takahashi K. , and Wakabayashi Sugawa S. , Synergistic Association of the Copper/Zinc Ratio Under Inflammatory Conditions With Diabetic Kidney Disease in Patients With Type 2 Diabetes: The Asahi Diabetes Complications Study, Journal of Diabetes Investigation. (2021) 13, no. 2, 299–307, 10.1111/jdi.13659. 34533892 PMC8847118

[16] Makhlough A. , Makhlough M. , Shokrzadeh M. , Mohammadian M. , Sedighi O. , and Faghihan M. , Comparing the Levels of Trace Elements in Patients With Diabetic Nephropathy and Healthy Individuals, Nephro-Urology Monthly. (2015) 7, no. 4, 10.5812/numonthly.28576, 2-s2.0-84940913342. PMC4628134 26539418

[17] Birney E. , Mendelian Randomization, Cold Spring Harbor Perspectives in Medicine. (2021) 12, no. 4. 10.1101/cshperspect.a041302 PMC9121891 34872952

[18] Yuan S. , Xu F. , Li X. , Chen J. , Zheng J. , Mantzoros C. S. , and Larsson S. C. , Plasma Proteins and Onset of Type 2 Diabetes and Diabetic Complications: Proteome-Wide Mendelian Randomization and Colocalization Analyses, Cell Reports Medicine. (2023) 4, no. 9, 10.1016/j.xcrm.2023.101174. PMC10518626 37652020

[19] Finan C. , Gaulton A. , Kruger F. A. , Lumbers R. T. , Shah T. , Engmann J. , Galver L. , Kelley R. , Karlsson A. , Santos R. , and Overington J. P. , The Druggable Genome and Support for Target Identification and Validation in Drug Development, Science Translational Medicine. (2017) 9, no. 383, 10.1126/scitranslmed.aag1166, 2-s2.0-85017477447. PMC6321762 28356508

[20] Zhao C. , Pan T. , Liu W. , Cheng F. , Zhao X. , Yu S. , Yang Y. , Zhang R. , and Sun W. , Causal Relationship Between Serum Zinc Levels and Diabetic Kidney Disease (DKD): A Plasma Proteomics Mediation Study, 2025, Biological Trace Element Research, 10.1007/s12011-025-04782-z. PMC12992405 40830297

[21] Fu S. , Qian M. , Yuan Z. , Su S. , Ma F. , Li F. , and Xu Z. , A New Perspective on Selenium′s Impact on Renal Function: European Population-Based Analysis of Plasma Proteome-Mediated Mendelian Randomization Study, Frontiers in Endocrinology. (2024) 15, 10.3389/fendo.2024.1410463. PMC11424436 39329105

[22] Evans D. M. , Zhu G. , Dy V. , Heath A. C. , Madden P. A. , Kemp J. P. , McMahon G. , St Pourcain B. , Timpson N. J. , and Golding J. , et alGenome-Wide Association Study Identifies Loci Affecting Blood Copper, Human Molecular Genetics. (2013) 22, no. 19, 3998–4006, 10.1093/hmg/ddt239, 2-s2.0-84881530978. 23720494 PMC3766178

[23] Sun B. B. , Maranville J. C. , Peters J. E. , Stacey D. , Staley J. R. , Blackshaw J. , Burgess S. , Jiang T. , Paige E. , Surendran P. , and Oliver-Williams C. , Genomic Atlas of the Human Plasma Proteome, Nature. (2018) 558, no. 7708, 73–79, 10.1038/s41586-018-0175-2, 2-s2.0-85048231672. 29875488 PMC6697541

[24] Kurki M. I. , Karjalainen J. , Palta P. , Sipilä T. P. , Kristiansson K. , Donner K. M. , Reeve M. P. , Laivuori H. , Aavikko M. , Kaunisto M. A. , and Loukola A. , FinnGen Provides Genetic Insights From a Well-Phenotyped Isolated Population, Nature. (2023) 613, no. 7944, 508–518, 10.1038/s41586-022-05473-8. 36653562 PMC9849126

[25] Burgess S. , Small D. S. , and Thompson S. G. , A Review of Instrumental Variable Estimators for Mendelian Randomization, Statistical Methods in Medical Research. (2017) 26, no. 5, 2333–2355, 10.1177/0962280215597579, 2-s2.0-85031678782. 26282889 PMC5642006

[26] Li F. , Liu Y. , Wang Z. , Zhao Q. , Li Y. , and Tang T. , A Mendelian Randomization Study With Populations of European Ancestry Rules Out a Causal Relationship Between Inflammatory Bowel Disease and Colorectal Cancer, Frontiers in Genetics. (2022) 13, 949325, 10.3389/fgene.2022.949325. 36092900 PMC9449310

[27] Burgess S. , Butterworth A. , and Thompson S. G. , Mendelian Randomization Analysis With Multiple Genetic Variants Using Summarized Data, Genetic Epidemiology. (2013) 37, no. 7, 658–665, 10.1002/gepi.21758, 2-s2.0-84885840876. 24114802 PMC4377079

[28] Burgess S. and Thompson S. G. , Interpreting Findings From Mendelian Randomization Using the MR-Egger Method, European Journal of Epidemiology. (2017) 32, no. 5, 377–389, 10.1007/s10654-017-0255-x, 2-s2.0-85019688623. 28527048 PMC5506233

[29] Bowden J. , Davey Smith G. , Haycock P. C. , and Burgess S. , Consistent Estimation in Mendelian Randomization With Some Invalid Instruments Using a Weighted Median Estimator, Genetic Epidemiology. (2016) 40, no. 4, 304–314, 10.1002/gepi.21965, 2-s2.0-84963670568. 27061298 PMC4849733

[30] Burgess S. , Davey Smith G. , Davies N. M. , Dudbridge F. , Gill D. , Glymour M. M. , Hartwig F. P. , Kutalik Z. , Holmes M. V. , and Minelli C. , et alGuidelines for Performing Mendelian Randomization Investigations: Update for Summer 2023, Wellcome Open Research. (2019) 4, 10.12688/wellcomeopenres.15555.3. PMC7384151 32760811

[31] Hemani G. , Tilling K. , and Davey Smith G. , Orienting the Causal Relationship Between Imprecisely Measured Traits Using GWAS Summary Data, PLoS Genetics. (2017) 13, no. 11, e1007081, 10.1371/journal.pgen.1007081, 2-s2.0-85036637858. 29149188 PMC5711033

[32] Fabregat A. , Jupe S. , Matthews L. , Sidiropoulos K. , Gillespie M. , Garapati P. , Haw R. , Jassal B. , Korninger F. , May B. , and Milacic M. , The Reactome Pathway Knowledgebase, Nucleic Acids Research. (2018) 46, no. D1, D649–d655, 10.1093/nar/gkx1132, 2-s2.0-85040929878. 29145629 PMC5753187

[33] Linder M. C. , Copper Homeostasis in Mammals, With Emphasis on Secretion and Excretion. A Review, International Journal of Molecular Sciences. (2020) 21, no. 14, 10.3390/ijms21144932. PMC7403968 32668621

[34] Goldstein I. M. , Kaplan H. B. , Edelson H. S. , and Weissmann G. , Ceruloplasmin: An Acute Phase Reactant That Scavenges Oxygen-Derived Free Radicals, Annals of the New York Academy of Sciences. (1982) 389, 368–379, 10.1111/j.1749-6632.1982.tb22150.x, 2-s2.0-0020332421. 6284006

[35] Jäger S. , Cabral M. , Kopp J. F. , Hoffmann P. , Ng E. , Whitfield J. B. , Morris A. P. , Lind L. , Schwerdtle T. , and Schulze M. B. , Blood Copper and Risk of Cardiometabolic Diseases: A Mendelian Randomization Study, Human Molecular Genetics. (2022) 31, no. 5, 783–791, 10.1093/hmg/ddab275. 34523676 PMC8895748

[36] Huang L. , Yang W. , Li L. , Feng X. , Cheng H. , Ge X. , Liu C. , Chen X. , Mo Z. , and Yang X. , Causal Relationships Between Blood Calcium, Iron, Magnesium, Zinc, Selenium, Phosphorus, Copper, and Lead Levels and Multisystem Disease Outcomes in Over 400,000 Caucasian Participants, Clinical Nutrition. (2022) 41, no. 5, 1015–1024, 10.1016/j.clnu.2022.02.020. 35390725

[37] Niu Y. Y. , Aierken A. , and Feng L. , Unraveling the Link Between Dietary Factors and Cardiovascular Metabolic Diseases: Insights From a Two-Sample Mendelian Randomization Investigation, Heart & Lung. (2024) 63, 72–77, 10.1016/j.hrtlng.2023.09.012. 37826923

[38] Brook M. , McCracken L. , Reddington J. P. , Lu Z. L. , Morrice N. A. , and Gray N. K. , The Multifunctional Poly(A)-Binding Protein (PABP) 1 Is Subject to Extensive Dynamic Post-Translational Modification, Which Molecular Modelling Suggests Plays an Important Role in Coordinating Its Activities, Biochemical Journal. (2012) 441, no. 3, 803–812, 10.1042/BJ20111474, 2-s2.0-84855911694. 22004688 PMC3298439

[39] Lemmer I. L. , Willemsen N. , Hilal N. , and Bartelt A. , A Guide to Understanding Endoplasmic Reticulum Stress in Metabolic Disorders, Molecular Metabolism. (2021) 47, 101169, 10.1016/j.molmet.2021.101169. 33484951 PMC7887651

[40] Krishnamoorthy L. , Cotruvo J. A. , Chan J. , Kaluarachchi H. , Muchenditsi A. , Pendyala V. S. , Jia S. , Aron A. T. , Ackerman C. M. , Wal M. N. , and Guan T. , Copper Regulates Cyclic-AMP-Dependent Lipolysis, Nature Chemical Biology. (2016) 12, no. 8, 586–592, 10.1038/nchembio.2098, 2-s2.0-84976274777. 27272565 PMC4955676

[41] Li F. , Wu X. , Liu H. , Zhang B. , Liu L. , and Li F. , Dietary Copper Supplementation Enhances Lipolysis in Rex Rabbits, Journal of Trace Elements in Medicine and Biology. (2021) 68, 10.1016/j.jtemb.2021.126851. 34464873

[42] Li H.-L. , Wu X. , Xu A. , and Hoo R. L.-C. , A-FABP in Metabolic Diseases and the Therapeutic Implications: An Update, International Journal of Molecular Sciences. (2021) 22, no. 17, 10.3390/ijms22179386. PMC8456319 34502295

[43] Zhong F.-F. , Wei B. , Bao G.-X. , Lou Y.-P. , Wei M.-E. , Wang X.-Y. , Xiao X. , and Tian J.-J. , FABP3 Induces Mitochondrial Autophagy to Promote Neuronal Cell Apoptosis in Brain Ischemia-Reperfusion Injury, Neurotoxicity Research. (2024) 42, no. 4, 10.1007/s12640-024-00712-4. 39008165

[44] Keller F. , Denicolò S. , Leierer J. , Kruus M. , Heinzel A. , Kammer M. , Ju W. , Nair V. , Burdet F. , Ibberson M. , and Menon R. , Association of Urinary Epidermal Growth Factor, Fatty Acid-Binding Protein 3, and Vascular Cell Adhesion Molecule 1 Levels With the Progression of Early Diabetic Kidney Disease, Kidney and Blood Pressure Research. (2024) 49, no. 1, 1013–1025, 10.1159/000542267. 39510044

[45] Xia X. , Hu Z. , Wang S. , and Yin K. , Netrin-1: An Emerging Player in Inflammatory Diseases, Cytokine & Growth Factor Reviews. (2022) 64, 46–56, 10.1016/j.cytogfr.2022.01.003. 35082104

[46] Ay E. , Marakoğlu K. , Kizmaz M. , and Ünlü A. , Evaluation of Netrin-1 Levels and Albuminuria in Patients With Diabetes, Journal of Clinical Laboratory Analysis. (2016) 30, no. 6, 972–977, 10.1002/jcla.21965, 2-s2.0-84963819785. 27076403 PMC6806737

[47] Townsend D. M. and Tew K. D. , The Role of Glutathione-S-Transferase in Anti-Cancer Drug Resistance, Oncogene. (2003) 22, no. 47, 7369–7375, 10.1038/sj.onc.1206940, 2-s2.0-0642374221. 14576844 PMC6361125

[48] Zecher M. , Guichard C. , Velásquez M. J. , Figueroa G. , and Rodrigo R. , Implications of Oxidative Stress in the Pathophysiology of Obstructive Uropathy, Urological Research. (2008) 37, no. 1, 19–26, 10.1007/s00240-008-0163-3, 2-s2.0-59449091512. 19082822

[49] Gutteridge J. M. and Halliwell B. , The Measurement and Mechanism of Lipid Peroxidation in Biological Systems, Trends in Biochemical Sciences. (1990) 15, no. 4, 129–135, 10.1016/0968-0004(90)90206-Q, 2-s2.0-0025369970. 2187293

[50] Uchida K. , 4-Hydroxy-2-Nonenal: A Product and Mediator of Oxidative Stress, Progress in Lipid Research. (2003) 42, no. 4, 318–343, 10.1016/S0163-7827(03)00014-6, 2-s2.0-0037411282. 12689622

[51] Yeh C. H. , Chiang H. S. , Lai T. Y. , and Chien C. T. , Unilateral Ureteral Obstruction Evokes Renal Tubular Apoptosis via the Enhanced Oxidative Stress and Endoplasmic Reticulum Stress in the Rat, Neurourology and Urodynamics. (2011) 30, no. 3, 472–479, 10.1002/nau.20855, 2-s2.0-79952768952. 21305585

[52] Hubatsch I. , Ridderstrom M. , and Mannervik B. , Human Glutathione Transferase A4-4: An Alpha Class Enzyme With High Catalytic Efficiency in the Conjugation of 4-Hydroxynonenal and Other Genotoxic Products of Lipid Peroxidation, Biochemical Journal. (1998) 330, no. 1, 175–179, 10.1042/bj3300175, 2-s2.0-0032519517. 9461507 PMC1219124

[53] Liang A. , Wang Y. , Woodard L. E. , Wilson M. H. , Sharma R. , Awasthi Y. C. , Du J. , Mitch W. E. , and Cheng J. , Loss of Glutathione S-Transferase A4 Accelerates Obstruction-Induced Tubule Damage and Renal Fibrosis, Journal of Pathology. (2012) 228, no. 4, 448–458, 10.1002/path.4067, 2-s2.0-84871222540. 22711583 PMC3760987

[54] Mandil R. , Prakash A. , Rahal A. , Koli S. , Kumar R. , and Garg S. K. , Evaluation of Oxidative Stress-Mediated Cytotoxicity and Genotoxicity of Copper and Flubendiamide: Amelioration by Antioxidants In Vivo and In Vitro, Toxicology Research. (2023) 12, no. 2, 232–252, 10.1093/toxres/tfad011. 37125329 PMC10141782

[55] Guo Q. , Kawahata I. , Cheng A. , Wang H. , Jia W. , Yoshino H. , and Fukunaga K. , Fatty Acid-Binding Proteins 3 and 5 Are Involved in the Initiation of Mitochondrial Damage in Ischemic Neurons, 102547Redox Biology. (2023) 59, 10.1016/j.redox.2022.102547. PMC9727700 36481733

[56] Singh S. R. , Jayakumar C. , Mohamed R. , Ranganathan P. V. , and Ramesh G. , Intracellular Kinases Mediate Increased Translation and Secretion of Netrin-1 From Renal Tubular Epithelial Cells, PLoS ONE. (2011) 6, no. 10, 10.1371/journal.pone.0026776, 2-s2.0-80055054103. PMC3202578 22046354

[57] Mohamed R. , Jayakumar C. , Ranganathan P. V. , Ganapathy V. , and Ramesh G. , Kidney Proximal Tubular Epithelial-Specific Overexpression of Netrin-1 Suppresses Inflammation and Albuminuria Through Suppression of COX-2-Mediated PGE2 Production in Streptozotocin-Induced Diabetic Mice, American Journal of Pathology. (2012) 181, no. 6, 1991–2002, 10.1016/j.ajpath.2012.08.014, 2-s2.0-84869155452. 23041393 PMC3509740

[58] Tak E. , Ridyard D. , Badulak A. , Giebler A. , Shabeka U. , Werner T. , Clambey E. , Moldovan R. , Zimmerman M. A. , Eltzschig H. K. , and Grenz A. , Protective Role for Netrin-1 During Diabetic Nephropathy, Journal of Molecular Medicine. (2013) 91, no. 9, 1071–1080, 10.1007/s00109-013-1041-1, 2-s2.0-84884256510. 23636509 PMC3766438

[59] van Bladeren P. J. , Glutathione Conjugation as a Bioactivation Reaction, Chemico-Biological Interactions. (2000) 129, no. 1-2, 61–76, 10.1016/S0009-2797(00)00214-3, 2-s2.0-0034534421. 11154735

[60] Bhattacharjee A. , Chakraborty K. , and Shukla A. , Cellular Copper Homeostasis: Current Concepts on Its Interplay With Glutathione Homeostasis and Its Implication in Physiology and Human Diseases, Metallomics. (2017) 9, no. 10, 1376–1388, 10.1039/c7mt00066a, 2-s2.0-85031777257. 28675215

[61] Maryon E. B. , Molloy S. A. , and Kaplan J. H. , Cellular Glutathione Plays a Key Role in Copper Uptake Mediated by Human Copper Transporter 1, American Journal of Physiology-Cell Physiology. (2013) 304, no. 8, C768–C779, 10.1152/ajpcell.00417.2012, 2-s2.0-84878653071. 23426973 PMC3625801

[62] Eaton D. L. and Bammler T. K. , Concise Review of the Glutathione S-Transferases and Their Significance to Toxicology, Toxicological Sciences. (1999) 49, no. 2, 156–164. 10416260 10.1093/toxsci/49.2.156

[63] Mannervik B. and Danielson U. H. , Glutathione Transferases--Structure and Catalytic Activity, CRC Critical Reviews in Biochemistry. (1988) 23, no. 3, 283–337. 3069329 10.3109/10409238809088226

