# Supplementary material for: The Link Between Serum Copper and Diabetic Kidney Disease: A Plasma Proteomic Perspective
Source: J Diabetes Res. 2026 Mar 16;2026:1950326. doi: 10.1155/jdr/1950326 (PMC13140866; doi:10.1155/jdr/1950326)
Supplement: Supplementary file 1 — Supporting Information 1 STROBE‐MR checklist. [file JDR-2026-1950326-s002.docx]

**STROBE-MR checklist of recommended items to address in reports of Mendelian randomization studies**^1^ ^2^

| **Item No.** | **Section** | **Checklist item** | **Page No.** | **Relevant text from manuscript** |
| --- | --- | --- | --- | --- |
| 1 | **TITLE and ABSTRACT** | Indicate Mendelian randomization (MR) as the study’s design in the title and/or the abstract if that is a main purpose of the study | 2 | This study employed Mendelian randomization (MR) analysis based on large-scale GWAS data to explore the causal relationship between serum copper levels and DKD. |
|  | **INTRODUCTION** |  |  |  |
| 2 | **Background** | Explain the scientific background and rationale for the reported study. What is the exposure? Is a potential causal relationship between exposure and outcome plausible? Justify why MR is a helpful method to address the study question | 5 | evidence suggests a relationship between serum zinc levels and copper's role in the development of DKD, leading to the proposal that the serum Cu/Zn ratio may serve as a marker for assessing DKD progression15. However, there is considerable variability in findings across studies7,16, with some reporting lower serum copper levels in DKD patients compared to the general population, while others fail to find any significant differences. This adds to the mystery of how copper plays a role in DKD. Such inconsistency further complicates the understanding of the relationship between serum copper and DKD. |
| 3 | **Objectives** | State specific objectives clearly, including pre-specified causal hypotheses (if any). State that MR is a method that, under specific assumptions, intends to estimate causal effects | 5-6 | Mendelian randomization (MR) is an epidemiological research method that uses genetic variation as an instrumental variable to explore whether exposure factors have a causal effect on health outcomes. In this study, based on large-scale GWAS data, we systematically explored the causal relationship between serum copper level and DKD using MR methods, and further mediated plasma proteomics to delve deeper into the mechanisms by which copper may contribute to the development of DKD. |
|  | **METHODS** |  |  |  |
| 4 | **Study design and data sources** | Present key elements of the study design early in the article. Consider including a table listing sources of data for all phases of the study. For each data source contributing to the analysis, describe the following: |  |  |
|  | a) | Setting: Describe the study design and the underlying population, if possible. Describe the setting, locations, and relevant dates, including periods of recruitment, exposure, follow-up, and data collection, when available. | 6 | Evans et al. published their summary GWAS data on serum copper levels in 2013. In their genome-wide association study, single nucleotide polymorphisms (SNPs) the effects of single nucleotide polymorphisms (SNPs) on blood copper concentrations were analyzed utilizing two adult cohorts from Australia and the United Kingdom, which included a total of 2,603 participants…… |
|  | b) | Participants: Give the eligibility criteria, and the sources and methods of selection of participants. Report the sample size, and whether any power or sample size calculations were carried out prior to the main analysis | 6 | Sun et al. presented summary GWAS data on plasma proteome levels in 2018. This study utilized an expanded version of aptamer-based multiplex protein assays to construct and query the genetic map of the human plasma proteome. The dataset quantified 3,282 plasma proteins in 3,301 healthy participants from 25 centers across England as part of the INTERVAL study, a genomic resource encompassing 50,000 blood samples…… |
|  | c) | Describe measurement, quality control and selection of genetic variants | 7 | Genome-wide testing was performed for 10.6 million imputed autosomal variants. The participants included individuals of European ancestry, both male and female, with SNP counts reaching up to 10,534,735. |
|  | d) | For each exposure, outcome, and other relevant variables, describe methods of assessment and diagnostic criteria for diseases | 7 | Additionally, genetic variant data related to health outcomes were sourced from the FinnGen project, a large-scale initiative designed to integrate genetic variation data with extensive health records from over 500,000 Finnish biobank samples. This project is a collaborative effort among Finnish research entities, biobanks, and international industry partners. |
|  | e) | Provide details of ethics committee approval and participant informed consent, if relevant | 7 | This project is a collaborative effort among Finnish research entities, biobanks, and international industry partners and all data underwent ethical review. |
| 5 | **Assumptions** | Explicitly state the three core IV assumptions for the main analysis (relevance, independence and exclusion restriction) as well assumptions for any additional or sensitivity analysis | 7-8 | To conduct MR analysis, certain crucial assumptions regarding instrumental variables must be met. Firstly, the instrumental variable must be significantly associated with the risk factor (relevance assumption). Secondly, the instrumental variable must be independent of any confounding factors (independence assumption). Thirdly, the instrumental variable should influence the outcome only through the risk factor and not via other pathways (exclusion restriction assumption). |
| 6 | **Statistical methods: main analysis** | Describe statistical methods and statistics used |  |  |
|  | a) | Describe how quantitative variables were handled in the analyses (i.e., scale, units, model) | 8 | Our analytical toolkit included the Inverse Variance Weighted (IVW), MR-Egger regression, Weighted Median (WM), Weighted Mode, and MR Robust Adjusted Profile Score (MR-RAPS). |
|  | b) | Describe how genetic variants were handled in the analyses and, if applicable, how their weights were selected | 9 | Based on the selected GWAS summary statistics, we established a series of criteria for screening SNPs. SNPs that were associated with the risk factor at the genome-wide significance level (p-value < 5 × 10⁻⁸) were initially chosen. For a more comprehensive analysis, in cases where no SNPs met this stringent threshold, we applied a more lenient genome-wide potential significance level (p-value < 1 × 10⁻⁵) as the cutoff. SNPs that were in linkage disequilibrium (LD) were removed using a threshold of r² < 0.001 and a window size of >10,000 kb. Relevant data for SNPs associated with the risk factor were extracted from the summary statistics of the outcome dataset. |
|  | c) | Describe the MR estimator (e.g. two-stage least squares, Wald ratio) and related statistics. Detail the included covariates and, in case of two-sample MR, whether the same covariate set was used for adjustment in the two samples | 10 | The F-statistic was calculated for each SNP to assess its strength as an instrumental variable; this statistic measures the strength of an SNP in explaining the risk factor. SNPs with an F-statistic < 10 were considered weak instruments and were therefore excluded from the analysis. An MR-PRESSO test was conducted to identify and eliminate SNPs with potential pleiotropy. The MR-Steiger test was applied to determine the causal direction of each SNP’s effect estimate, and SNPs with incorrect directions were excluded. |
|  | d) | Explain how missing data were addressed | 8 | Finally, PhenoScanner was used to assess the potential associations of each SNP with confounding factors, and SNPs potentially violating the independence assumption were removed. |
|  | e) | If applicable, indicate how multiple testing was addressed | Not available | Not available |
| 7 | **Assessment of assumptions** | Describe any methods or prior knowledge used to assess the assumptions or justify their validity | 8 | Based on the selected GWAS summary statistics, we established a series of criteria for screening SNPs. SNPs that were associated with the risk factor at the genome-wide significance level (p-value < 5 × 10⁻⁸) were initially chosen. For a more comprehensive analysis, in cases where no SNPs met this stringent threshold, we applied a more lenient genome-wide potential significance level (p-value < 1 × 10⁻⁵) as the cutoff. SNPs that were in linkage disequilibrium (LD) were removed using a threshold of r² < 0.001 and a window size of >10,000 kb. |
| 8 | **Sensitivity analyses and additional analyses** | Describe any sensitivity analyses or additional analyses performed (e.g. comparison of effect estimates from different approaches, independent replication, bias analytic techniques, validation of instruments, simulations) | 8 | Pleiotropy, a phenomenon where a single gene influences multiple traits, can be categorized into horizontal and vertical forms. While vertical pleiotropy does not typically affect the validity of study outcomes, horizontal pleiotropy poses a significant threat to the reliability of conclusions and must be addressed. |
| 9 | **Software and pre-registration** |  |  |  |
|  | a) | Name statistical software and package(s), including version and settings used | 12 | To facilitate the visualization in the conclusion section, scatter plots were generated for each SNP, illustrating the relationship between the exposure effects and the outcome effects, accompanied by regression curves to demonstrate causal estimates. A significance heatmap for MR analysis was meticulously crafted to present the results. Funnel plots were employed to evaluate potential directional pleiotropy and assess the distribution of the data. The final causal estimates were utilized to generate forest plots, which presented the results for each SNP and the overall MR analysis. All statistical analyses in this study were conducted using R software (version 4.2.3) and the R packages TwoSampleMR, MRP-RESSO, and mr.raps. |
|  | b) | State whether the study protocol and details were pre-registered (as well as when and where) | Not available | Not available |
|  | **RESULTS** |  |  |  |
| 10 | **Descriptive data** |  |  |  |
|  | a) | Report the numbers of individuals at each stage of included studies and reasons for exclusion. Consider use of a flow diagram | 12 | The SNPs that exhibited a strong correlation with serum copper levels were predominantly mapped to chromosome 1 (Figure 2-A). Among these, SNPs rs2769264 and rs1175550 reached genome-wide significance, with mutations in these SNPs consistently promoting elevated serum copper levels (Figure 2-B). In contrast, the SNPs strongly associated with DKD were mainly located on chromosome 6 (Figure 3-A). Among the top five genome-wide significant SNPs, rs9273363, rs9270891, rs59377618, rs564782870, and rs11967629 were identified. Mutations in these SNPs were shown to significantly contribute to the development of DKD (Figure 3-B). Details regarding the SNPs included in the MR study are presented in Supplementary Table 2. |
|  | b) | Report summary statistics for phenotypic exposure(s), outcome(s), and other relevant variables (e.g. means, SDs, proportions) | Supplementary table 2 | Supplementary table 2 |
|  | c) | If the data sources include meta-analyses of previous studies, provide the assessments of heterogeneity across these studies | Not available | Not available |
|  | d) | For two-sample MR:  i.  Provide justification of the similarity of the genetic variant-exposure associations between the exposure and outcome samples  ii.  Provide information on the number of individuals who overlap between the exposure and outcome studies | i. Supplementary table 2  ii.  12 | i. Supplementary table 2  ii.   Based on the information from the data set sources, there is no sample overlap in the MR analysis of this study. |
| 11 | **Main results** |  |  |  |
|  | a) | Report the associations between genetic variant and exposure, and between genetic variant and outcome, preferably on an interpretable scale | Supplementary table 3 | Supplementary table 3 |
|  | b) | Report MR estimates of the relationship between exposure and outcome, and the measures of uncertainty from the MR analysis, on an interpretable scale, such as odds ratio or relative risk per SD difference | 13-14 | In the MR analysis, elevated serum copper levels were found to promote the development of DKD (OR [95% CI]: 1.123 [1.076-1.173], P < 0.001), with no evidence of heterogeneity or pleiotropy detected |
|  | c) | If relevant, consider translating estimates of relative risk into absolute risk for a meaningful time period | Not available | Not available |
|  | d) | Consider plots to visualize results (e.g. forest plot, scatterplot of associations between genetic variants and outcome versus between genetic variants and exposure) | Figures 2-3 | Figures 2-3 |
| 12 | **Assessment of assumptions** |  |  |  |
|  | a) | Report the assessment of the validity of the assumptions | Supplementary table 4-6 | Supplementary table 4-6 |
|  | b) | Report any additional statistics (e.g., assessments of heterogeneity across genetic variants, such as *I^2^*, Q statistic or E-value) | Supplementary table 4-6 | Supplementary table 4-6 |
| 13 | **Sensitivity analyses and additional analyses** |  |  |  |
|  | a) | Report any sensitivity analyses to assess the robustness of the main results to violations of the assumptions | Supplementary table 4-6 | Supplementary table 4-6 |
|  | b) | Report results from other sensitivity analyses or additional analyses | Supplementary table 4-6 | Supplementary table 4-6 |
|  | c) | Report any assessment of direction of causal relationship (e.g., bidirectional MR) | Not available | Not available |
|  | d) | When relevant, report and compare with estimates from non-MR analyses | Not available | Not available |
|  | e) | Consider additional plots to visualize results (e.g., leave-one-out analyses) | Figures 2-3 | Figures 2-3 |
|  | **DISCUSSION** |  |  |  |
| 14 | **Key results** | Summarize key results with reference to study objectives | 15 | In this study, based on large-scale GWAS data, we systematically explored the causal relationship between serum copper level and DKD using MR methods, and found that an increase in serum copper level would lead to an increased risk of DKD. These results suggest that targeted interventions, such as rational copper supplementation and dietary adjustments, could be employed to mitigate the risk of DKD in high-risk populations. |
| 15 | **Limitations** | Discuss limitations of the study, taking into account the validity of the IV assumptions, other sources of potential bias, and imprecision. Discuss both direction and magnitude of any potential bias and any efforts to address them | 19-20 | This study acknowledges several limitations that warrant consideration. Firstly, the GWAS data concerning serum copper levels were obtained from two cohorts of adults located in Australia and the United Kingdom. Previous research has indicated variability in serum copper levels across different population, which may be attributed to factors such as ethnicity, diet, and local environmental conditions. Consequently, the generalizability of our findings might be restricted. Secondly, the GWAS data on plasma proteome levels was derived from a population of European ancestry, further limiting the applicability of our results to the broader population. Additionally, our analysis relied on publicly available summary statistics, and raw clinical outcome data for individual participants were not accessible. This constraint hindered our ability to conduct further population stratification analyses. Lastly, although we conducted a comprehensive examination of various plasma proteins and pathways involved in DKD, we did not experimentally validate this, and the specific causal effects of this need to be explored in further studies. |
| 16 | **Interpretation** |  |  |  |
|  | a) | Meaning: Give a cautious overall interpretation of results in the context of their limitations and in comparison with other studies | 15 | In diabetic patients, copper metabolism is often disrupted, and this dysregulation is increasingly recognized as a significant contributor to the progression of ESRD, with approximately 40% of diabetic patients developing DKD3. Current therapeutic options are limited to renal replacement therapy and symptomatic supportive care. Therefore, exploring the potential role of copper in the development of DKD is important for future clinical interventions in DKD. In this study, based on large-scale GWAS data, we systematically explored the causal relationship between serum copper level and DKD using MR methods, and found that an increase in serum copper level would lead to an increased risk of DKD. These results suggest that targeted interventions, such as rational copper supplementation and dietary adjustments, could be employed to mitigate the risk of DKD in high-risk populations. |
|  | b) | Mechanism: Discuss underlying biological mechanisms that could drive a potential causal relationship between the investigated exposure and the outcome, and whether the gene-environment equivalence assumption is reasonable. Use causal language carefully, clarifying that IV estimates may provide causal effects only under certain assumptions | 15 | To further investigate the mechanism of elevated copper level in the development of DKD, we identified eleven pathways involved in the enrichment analysis, including Netrin-1 signaling, Glutathione conjugation and so on. As mentioned above, the levels of Netrin-1 increase concomitantly with elevated copper levels, and numerous studies have proposed Netrin-1 as a potential biomarker for kidney injury…… |
|  | c) | Clinical relevance: Discuss whether the results have clinical or public policy relevance, and to what extent they inform effect sizes of possible interventions | 18 | But interestingly, our MR study found that increased serum copper level led to an increased risk of DKD, suggesting that serum copper may contribute to DKD progression through renal damage pathways independent of blood glucose levels. |
| 17 | **Generalizability** | Discuss the generalizability of the study results (a) to other populations, (b) across other exposure periods/timings, and (c) across other levels of exposure | 20 | Secondly, the GWAS data on plasma proteome levels was derived from a population of European ancestry, further limiting the applicability of our results to the broader population....... |
|  | **OTHER INFORMATION** |  |  |  |
| 18 | **Funding** | Describe sources of funding and the role of funders in the present study and, if applicable, sources of funding for the databases and original study or studies on which the present study is based | 21 | This study was supported by Natural Science Foundation of Jilin Province (No. YDZJ202201ZYTS126) and Natural Science Foundation of Jilin Province-Talent Specialization-Youth Growth Science and Technology Project (No. 20240602091RC). |
| 19 | **Data and data sharing** | Provide the data used to perform all analyses or report where and how the data can be accessed, and reference these sources in the article. Provide the statistical code needed to reproduce the results in the article, or report whether the code is publicly accessible and if so, where | 20 | All original contributions presented in this study are included within the article and its Supplementary Material. Additional inquiries can be addressed to the corresponding author. |
| 20 | **Conflicts of Interest** | All authors should declare all potential conflicts of interest | 20 | The authors declare that the research was conducted in the absence of any commercial or financial relationships that could be construed as a potential conflict of interest. |

This checklist is copyrighted by the Equator Network under the Creative Commons Attribution 3.0 Unported (CC BY 3.0) license.

1. Skrivankova VW, Richmond RC, Woolf BAR, Yarmolinsky J, Davies NM, Swanson SA, et al. Strengthening the Reporting of Observational Studies in Epidemiology using Mendelian Randomization (STROBE-MR) Statement. JAMA. 2021;under review.

2. Skrivankova VW, Richmond RC, Woolf BAR, Davies NM, Swanson SA, VanderWeele TJ, et al. Strengthening the Reporting of Observational Studies in Epidemiology using Mendelian Randomisation (STROBE-MR): Explanation and Elaboration. BMJ. 2021;375:n2233.
